# Effects of pericytes and colon cancer stem cells in the tumor microenvironment

**DOI:** 10.1186/s12935-019-0888-9

**Published:** 2019-07-01

**Authors:** Elsa N. Garza Treviño, Paulina Delgado González, Carlos I. Valencia Salgado, Alejandra Martinez Garza

**Affiliations:** Department of Biochemistry and Molecular Medicine, School of Medicine, Autonomous University of Nuevo León, Monterrey, Mexico

**Keywords:** Cancer stem cells, Pericytes, CRC treatment, Chemoresitance

## Abstract

Colorectal cancer (CRC) is one type of tumor with the highest frequency and mortality worldwide. Although current treatments increase patient survival, it is important to detect CRC in early stages; however, most CRC, despite responding favorably to treatment, develop resistance and present recurrence, a situation that will inevitably lead to death. In recent years, it has been shown that the main reason for drug resistance is the presence of colon cancer stem cells (CSC). Pericytes are also capable of tumor homing and are important cellular components of the tumor microenvironment (TME), contributing to the formation of vessels and promoting metastasis; however, they have not been considered very important as a therapeutic target in cancer. In this review, we highlight the contribution of pericytes and cancer stem cells to some classical hallmarks of cancer, namely, tumor angiogenesis, growth, metastasis, and evasion of immune destruction, and discuss therapies targeting pericytes and cancer stem cells in CRC.

## Background

Colorectal cancer (CRC) is a major cause of morbidity and mortality throughout the world. It is the third most common cancer worldwide and the most common malignant tumor in the lower digestive tract [1]. The populations of cells that make up a cancer are manifestly heterogeneous at the genetic, epigenetic, and phenotypic levels. Predominant cell types include immune cells, fibroblasts, adipocytes, endothelial cells (ECs), mesenchymal stroma/cancer stem cells (CSC) and pericytes [2].

The response to treatment is affected by the complexity and immune diversity within the tumor microenvironment (TME) [3]. Immune cell infiltration is a predictive factor in primary tumors, which correlates with tumor mass reduction and patient survival. There is a great interpersonal variability in the same kind of tumor with infiltrating immune cells, including effector T lymphocytes (CTLs), T-helper (TH) cells, T-regulatory cells (T-reg), B cells, natural killer (NK) cells, dendritic cells (DCs) cells, macrophages, myeloid derived suppressor cells (MDSC), and granulocytes [4]. Also, recent studies in CRC have attributed a good prognosis to infiltration by Th1 cells, M1 macrophages, dendritic cells and NK cells, while the presence of M2 macrophages, MDSCs, Th17 and B cells has been associated with a poor outcome [4].

The main mechanisms that eliminate tumor cells in CRC are gamma IFN and TNF (α and β) producing CD4 + TH1 cells and IL10 secreted by FoxP3^+^ regulatory T cells by NK or γδ T cells that suppress or downregulate induction and proliferation of effector T cells at the tumor site [5, 6]. Cancer-associated fibroblasts (CAFs) are the dominant cell type within the reactive stroma of many tumor types like CRC. This promotes invasiveness by secreting metalloproteinase as CXCL12, which activates CXCL12/CXCR4 signaling [7]. Growth factors, such as transforming growth factor beta (TGF-β), platelet-derived growth factor (PDGF), and fibroblast growth factor (FGF), released by tumor cells, are key mediators of CAF activation and contribute markedly to self-renewal of CSC and the development of chemotherapy drug resistance (by secreting TGF-β1). Adipocytes in obesity can actively secrete multiple adipokines and cytokines such as leptin, adiponectin, IL-6, MCP-1 (monocyte chemoattractant protein 1), and TNF-α which are proinflammatory signals [8]. Over time, chronic inflammation can cause DNA damage and promote cancer growth and metastasis. Macrophages contribute as growth tumor cells by inducing formation of new blood vessels from existing ones; this is called angiogenesis. Tumor angiogenesis not only provides the tumor cells with nutrients and oxygen and allows removal of metabolic wastes, but also presents the metastatic tumor cells with points of entry to the circulatory system. Some proangiogenic factors, such as vascular endothelial growth factor (VEGF) and stromal cell-derived factor 1 (SDF-1) promote the repair of injured vascular endothelial cells and neovascularization. Some studies have shown that CXCL12 promotes the synthesis and secretion of VEGF, and CXCL12 combined with VEGF enhances ischemic angiogenesis [9].

Efforts to profile tumor-infiltrating immune cells often have inherent limitations in sample availability, great interpersonal variability, and technological capability, thus restricting research into the local immune response. Therefore, tumor recurrence and metastasis are two critical survival-influencing factors of CRC [10].

Many researchers have observed that some cancer cells acquire the characteristics of cancer stem cells (CSC) through epithelial–mesenchymal transition (EMT), which is responsible for promoting invasion, metastasis and chemotherapy and radiotherapy resistance [11]. Furthermore, successful development of extravasation depends on pericyte cells and signals from the niche in the TME.

The purpose of this article is to highlight the importance of CSC and pericytes in the TME as principal microRNAs innovative therapeutic strategies that can be used for CRC.

### Cancer stem cells

Tumor-initiating cells or cancer stem cells (CSCs) are a subpopulation in tumor tissue that are distinct from non-malignant stem cells. CSCs possess unique characteristics such as self-renewal and diferentiation cloning to lineages inside epithelial tissue, giving them great heterogeneity [12]. This can be reflected in the intra-tumoral histological variability recognized a few years ago. They express detoxifying enzymes or efflux bombs that have high efficacy for drug molecule extrusion outside cells; providing them with resistance mechanisms against chemotherapy and radiotherapy. Aside from their high efficiency to generate tumors, slow growth rate, homing and treatment resistance are main characteristics responsible for recurrence and metastasis [13]. In normal intestinal tissue growth, the signaling pathways, Wingless/Int (WNT), Hedgehog (Hh), and Notch, are considered the most important regulators of stemness maintenance and self-renewal [14]. However, aberrant activation of these pathways serve as signaling pathways for the maintenance and proliferation of CSC in tumorigenesis [15, 16].

For CSC stemness maintenance, WNT promotes transcription of NANOG, OCT4, KLF4, EGFR, and LGR5 (GPR49). A Lgr5^+^CD44^+^EpCAM^+^ subpopulation could generate more colonies than any other subpopulation, indicating a higher tumorigenic potential that can produce metastatic disease and strictly defines as markers CSC in human CRC [17]. Aberrant activation of Notch protects CSCs from apoptosis via inhibition of the cell cycle kinase inhibitor p27 as well as ATOH1, a transcription factor [18]. Fender et al. suggested that Notch-1 can increase expression of the EMT/stemness-associated proteins, CD44, Slug, Smad-3, and induce Jagged-1 (Jag-1) expression by increased migration and increased anchorage independent growth [19]. In colon cancer, Notch activation in cancer cells by adjacent blood vessel cells increases trans-endothelial migration, and therefore, metastasis [20]. The expression of Jag1 by ECs activates Notch signaling in local pericyte precursor cells to induce pericyte differentiation [21]. Also, WNT and Hh signaling frequently operate in unison to control cell growth, development, and tissue homeostasis of normal and neoplastic stem cells by regulating gene transcription of VEGF, cMyc, Nanog, Sox2 and Bmil. The Hh pathway controls expression of ABC transporter proteins such as multi-drug resistance protein-1, leading to chemoresistance of CSCs, which effects survival, EMT, metastasis, and CSC expansion [22]. For a more detailed review of the mechanisms involved in these routes, we recommend works by Zhan et al. for WNT [23], Skoda et al. for Hh [24] and Brzozowa et al. for Notch [25].

The discovery of CSC antigens is not based on the overexpression of typical tumor antigens but on the presence of antigens in populations of cells that have stem cell-like properties. However, it is important to note that variable expression levels of antigens on CSCs and their frequent coexpression on normal stem cells have made CSC antigen distinction difficult (Lgr5, CD44, CD24, CD26, CD29, CD166, CD326, CD133, EpCAM and ALDH). LGR5^+^ CSCs are required for the maintenance of established liver metastases [26].

Three genes, OCT4, SOX2 and NANOG, play a dominant role in regulating pluripotency and are known to influence stem cell maintenance, tumor growth, invasion, EMT and metastasis. However, SALL4 was recognized recently as a zinc finger transcriptional factor regulating multiple targeted genes (OCT4, SOX2, and KLF4, Bmi-1 and PTEN). SALL4 is capable of stimulating Wnt/β-catenin signaling by directly binding to β-catenin and functioning as an oncogene in diverse tumors (leukemia, liver cancer, breast cancer, gastric and CRC). Previously, SALL4 mRNA levels in the blood were found to be significantly higher in patients with CRC than in control subjects, but lower in patients with a local cancer than in those with invasive CRC [27].

The remarkable complexity that involves cancer from the point of view of colon stem cells can be observed by the large number of markers they have and how their expression is modified depending on the factors that are exposed inside and outside of the TME. CRC develops as a result of serial alterations in oncogenes and tumor suppressor genes (APC, KRAS and TP53) [28]. However, recent studies reported that hypoxia-associated cell-type plasticity and epigenetic alterations can deregulate fundamental signaling pathways controlling self-renewal and differentiation, including Wnt, Notch, Myc and Hh pathways, contributing to this CSC heterogeneity and the potential implications for generating metastasis by EMT [29, 30].

### Epithelial–mesenchymal transition (EMT)

The normal transition of colon or rectum mucosal cells from epithelial to mesenchymal (EMT) cells regulates healthy intestinal architecture and also defines the balance between proliferation and differentiation mediated by the WNT pathway. CRC mutations in the APC gene (present in 80% of sporadic cancers) result in constant activation of the Wnt pathway (β-catenin), promoting the transition to the mesenchymal phenotype [31]. It is considered that during this transition process, a mechanism is activated where tumor (epithelial) cells lose their polarity as well as adhesion mediated by E-cadherin downregulation of other epithelial genes, components of the tight junctions; this includes members of the claudin family and cytokeratins, which produce the reorganization of the cytoskeleton. Also, during this process, the basement membrane and the extracellular matrix are destroyed by secretion of enzymes such as matrix metalloproteinase, which cause cells to pass from an adherent epithelial phenotype to a non-adherent mesenchymal phenotype [32]. Therefore, the phenotype fibroblast-like cell of non-adherent cells into spindle-shaped which characteristically upregulate mesenchymal markers; e.g., vimentin, N-cadherin and fibronectin which are associated with invasion of adjacent tissues and the formation of metastases [33]. Metastases originate because non-adherent cells circulate through the lymphatic and vascular blood systems which, in the final analysis, contribute to the intra- or extravasation of the transformed cells [31, 32].

The EMT process is regulated by TGF-β. This signal induces the expression of other growth factors such as fibroblast specific protein (FSP1), smooth muscle alpha actin (SMAα), vascular endothelial growth factor (VEGF) and the cytokines, IL-6, IL-23 and/or IL-1β (pro-inflammatory) from CD4^+^ T lymphocytes, which participate in maintaining a microenvironment to promote this complex process. In addition, the activation of transcription factors such as Snail1/2, Slug, Twist1 and Zeb1/2 and pathways such as Wnt, Hedgehog (HH), bone morphogenic protein (BMP), Notch, and platelet-derived growth factor (PDGF), Oct4 and Sox2, are involved in uncontrolled proliferation, regulate downexpression of E-cadherin, and proteases that promote loss of cell adhesion and stemmess phenotype [34, 35].

Recent studies suggest that MSCs induce EMT in colon cancer cells via direct cell-to-cell contact or indirect communication between MSC-derived exosomes which may play an important role in colon cancer metastasis. Also, in human CRC, EMT enhances the migratory and invasive properties of cancer cells which results in invasive lesions and tumor peripheries at the interface between cancer cells and host cells surrounded by ECM [36].

### Pericytes

Pericytes are specialized mesenchymal cells present at intervals along the walls of capillaries (and post-capillary venules), which vary greatly in morphology and marker expression in different tissues [37]. Mesenchymal stem cells and pericytes display remarkable similarities in terms of their marker expression, their ability to self-renew, and their potential to differentiate into multiple cell types such as adipocytes, chondrocytes, osteocytes and myocytes in culture.

Furthermore, some pericyte markers are PDGFR-β (platelet-derived growth factor receptor-beta), NG2 (chondroitin sulfate proteoglycan 4), CD13 (alanyl (membrane) aminopeptidase), αSMA (alpha-smooth muscle actin) [38], Desmin and CD146, are not uniquely found on pericytes but are also expressed on other cell types, most notably endothelial and smooth muscle cells, and are often dynamically expressed [39, 40]. Recent studies have shown that CD146 is constitutively expressed in the pericytes of several organs and functions as a component of endothelial junctions to reduce the paracellular permeability of peripheral endothelial cells. CD146 (also known as MCAM, S-endo-1, P1H12, and MUC18) was identified as a novel endothelial biomarker for angiogenesis in the tumor progression of several malignancies. CD146 is a potential marker for the diagnosis of malignancy in cervical and endometrial cancer, including melanoma and lung cancer [41, 42].

Pericytes residing in different tissues have been termed according to their function and morphology, such as hepatic stellate cells in the liver and glomerular mesangial cells in the kidney. The morphology of pericytes can be stellate or spindle-like with finger-like projections surrounding the vessels which are now believed to have a role in regulating blood flow and inflammatory cell trafficking [43]. Under pathological conditions, pericytes may differentiate into myofibroblasts, contributing to kidney fibrosis [44].

Pericytes are involved in the preservation of vascular stability and homeostasis, including regulation of blood flow, structural maintenance of the vasculature, vascular permeability, and remodeling of ECM [45]. Emerging evidence demonstrated that pericytes are an important cellular component in the TME associated with angiogenesis, metastasis, resistance to treatment and patient mortality; however, the mechanisms are poorly understood [44].

**Fig. 1:**
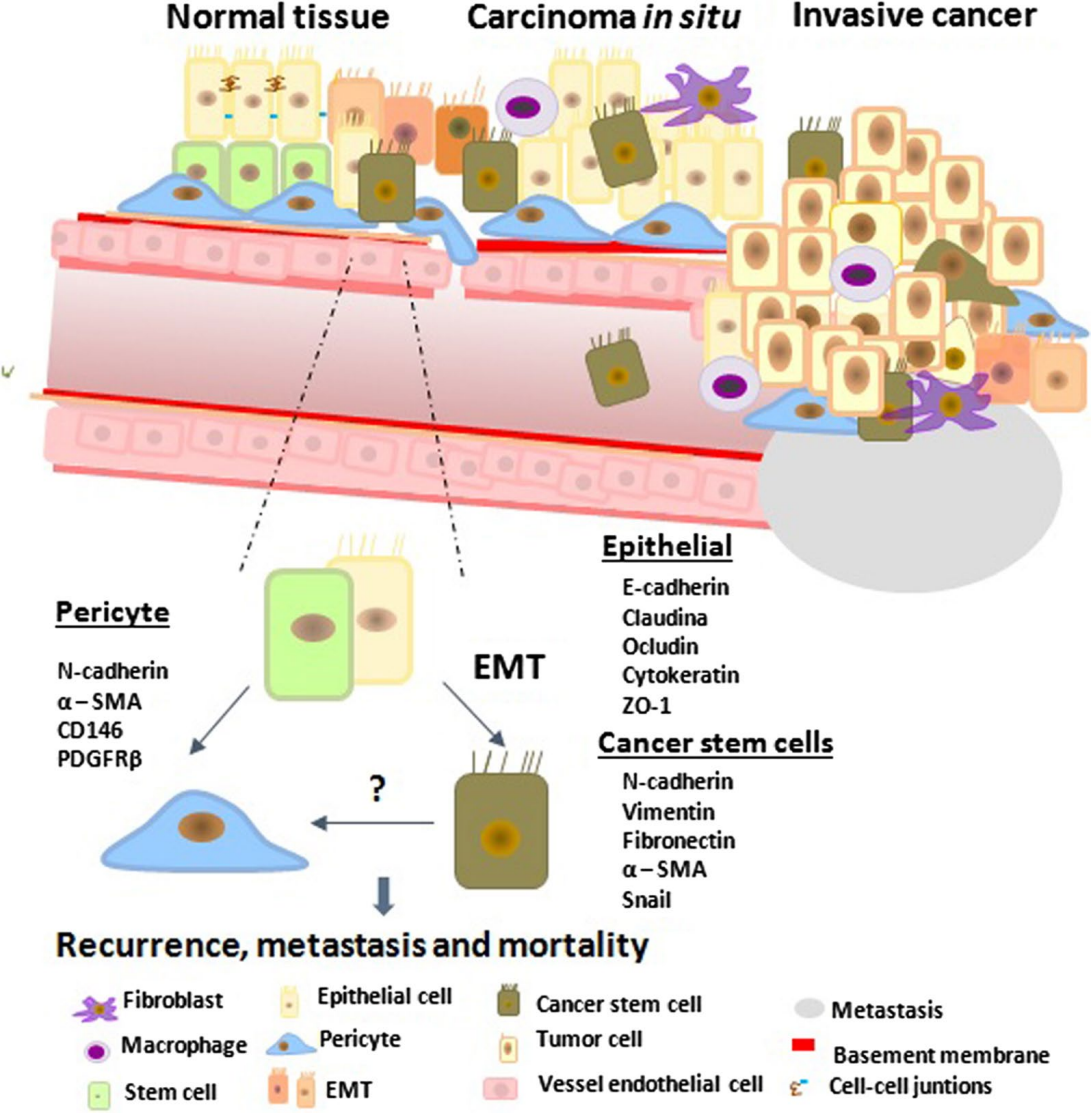
Interaction pericytes and cancer stem cells. Tumorigenesis activates EMT-promoting transcription factors (TWIST, SNAIL and ZEB) through pathways known to play critical as WNT, NOTCH, TGF-β and NF-κB cascades and hypoxia. Cancer stem cells were recently found to function as pericyte progenitors thus reciprocal interaction between pericytes and CSC is highly beneficial to tumor development, contributing to tumor angiogenesis and metastasis

Endothelial cells (ECs) that line the inner surface of vessels, directly participate in oxygen delivery, nutrient supply, and removal of waste products. During blood vessel maturation, endothelial cells (ECs) secrete platelet-derived growth factor (PDGF), which chemoattracts pericytes that express PDGFRβ. The ligand binding with the receptor provides vessel stability. VEGF produced by endothelial cells is crucial for normal vascular homeostasis. It is known that during EMT, PDGFR is expressed by stromal cells of mesenchymal origin, such as pericytes, which derive mainly from the cephalic region and the neural crest [46] From EMT, mesothelial cells attach to the pericytes of the intestine, liver, heart and lung. This is very important since during tumor development, some tumor cells after EMT suffer a loss of junctions from neighboring cells, diminishing expression of E-cadherin; also, high levels of PDGFR can begin to express markers similar to pericytes (NG2 and SMA) [47, 48]. This represents the epithelial transition to pericytes (EPT), a process induced by TGF-β, which may also activate the EMT program as well as contribute to the development of both normal and tumor pericytes (Fig. 1). In this way, some tumor cells are recruited or differentiated to pericytes to help vascularize tumor tissue and intratumoral vasculature, promoting metastasis [49]. These malignant pericytes may further acquire properties that promote their mobility and invasiveness during tumor metastasis [50]. Thus, malignant pericytes may be of central importance for both tumor angiogenesis and tumor metastasis [51].

Angiogenesis involves the formation of new vessels to supply nutrients to the tumor, promoting cancer survival, growth, and dissemination. This complex process is regulated through ECs and pericytes that express high levels of PDGF and VEGF/VEGFR (receptor tyrosine kinases such as VEGFR1, VEGFR2, and VEGFR3). Factors are involved in stimulating tumor angiogenesis indirectly by inducing VEGF, TGF-α and β, TNF-α, keratinocyte growth factor, insulin-like growth factor I (IGF-I), FGF, PDGF, and cytokines [interleukin (IL)-1α and IL-6 and EGF on tumor cells]. EGF, a key EGFR ligand, is one of the many growth factors that drive VEGF expression. EGFR is one of four members of the HER/erbB family of receptor tyrosine kinases [HER1 (EGFR/erbB1), HER2 (neu, erbB2), HER3 (erbB3), and HER4 (erbB4)] that is present on all epithelial and stromal cells, and on many smooth muscle cells; however, EGFR overexpression and aberrant EGFR expression has been observed in numerous tumor cell correlates with increased proliferative, angiogenic activity, and poor prognosis [52]. Increased proliferation and angiogenesis by EGFR are thought to be caused by the binding ligands TGFα and EGF, which have shown to function as chemoattractants for endothelial cells and promote the expression of VEGF by tumor cells. Many observations indicate that anti-angiogenic therapy may have limited efficacy, and in most patients, the cancers eventually display resistance to this treatment. Previous studies have shown that this resistance mechanism is associated with hypoxia-induced alterations. Tumor cell deprivation of oxygen induces HIF1α which dimerizes with HIF1β and translocates to a nucleus where transcription regulates expression of genes, such as VEGF, PDGF, bFGF, erythropoietin, angiopoietin, and placental growth factor (PIGF) which increase cell proliferation, metabolism, and abnormal tumor blood vessels [53]. Activation of EMT is a molecular pathway that evades therapeutic efficiency and produces resistance to anti-angiogenic therapy. During this process, a few CSCs, using the EPT, give rise to cancer cells that function as pericytes to stabilize blood vessels. Migration of CSC to blood vessels in the primary tumor is a natural part of the intravasation process, which depends on EMT and EPT produced signals that coordinate to generally enable cancer cells to be chemoattracted or associated with ECs, and help stabilize the vasculature or intravasate for metastasis.

The capacity of CSCs to generate vascular pericytes allows active vascularization in CRC to support tumor growth [54]. Therefore, we believe that pericytes may have a crucial role in mediating therapeutic resistance in CRC. Several studies of pericyte and tumor development were mostly focused on angiogenesis, showing that blockage of pericyte recruitment or function leads to reduced tumor growth due to compromised vessel structure and extravasation tumor cells [47]. Also, poor pericyte coverage has also been confirmed to have a correlation with a worst prognosis for patients with cancer that originates leaky vessels that increase intratumoral/interstitial plasma volume and elevate local pressure contributing to the progression and metastasis in the tumor by releasing factors that affect tumor invasion. High vascular density at the CRC invasion front is directly associated with recurrence, metastasis, and patient mortality. Ultimately, pericyte-targeted therapies should be tested in combination with other treatment modalities to address possible synergistic effects avoiding metastatic spread [55]. Hsu et al. [56] recently demonstrated in patients with metastasic CRC with wild-type KRAS exon 2, who had received cetuximab (anti-EGFR) and then bevacizumab (anti-VEGF), and standard chemotherapy, an increased overall survival by reductions in microvasculature density and tumor metastasis. The principle of first blocking EGFR is based on eliminating the vasculature that promotes tumor growth after that the tumor cells become more susceptible to being eliminated by antiangiogenic therapy. Until now the use of antiangiogenic agents is far from being effective in CRC since resistance to these treatments occurs mainly through the EMT and EPT routes. We believe that this additive effect in the treatment of CRC should be addressed not only in CSC but also in pericytes and this is why we review the main therapeutic targets in CRC.

## Therapeutic strategy

First-line treatment in patients with CRC is FOLFOX, which includes 5-fluorouracil (5-FU), oxaliplatin, and leucovorin. However, most patients develop resistance to this treatment and die within 1–10 years after its initiation [57]. Angiogenesis is required for invasive tumor growth and metastasis, which is mediated through VEGF and EGFR. Patients with metastatic CRC are currently treated with irinotecan and immunotherapy (bevacizumab, ramucirumab, and Ziv-aflibercept against VEGF and either cetuximab or panitumumab against EGFR) [58] DJ-1 (PARK7/CAP1/RS) is a multifunctional protein that protects neurons from oxidative stress by activating Akt/mTOR, MEK/ERK, NF-κB, and HIFα signaling pathways. Overexpression of DJ-1 in many tumor types correlated with promoting cancer cell survival, proliferation, and metastasis. Results recently suggest that DJ-1 is a potential prognostic and therapeutic target in invasive CRC [59]. More recently, the DART protein MGD007 was designed to co-engage T lymphocytes with CRC cells through the cell surface antigens, CD3 and gpA33, respectively, in order to promote T-cell recruitment and anti-tumor activity [60]. In addition, novel 89Zr-labeled anti-LGR5 mAbs were developed for evaluating the imaging potential of the CSC marker and were useful for stratifying patients that would respond best to an LGR5-targeted ADC therapy, and for monitoring treatment response in CRC [61]. Targeting strategies in self-renewal pathways in CSCs, including their pharmacological antagonists Hh ligand Inhibitors (PTCH1 inhibitor or RU-SKI [62]. GLI Antagonists (TAK-441-trial advanced CRC), SMO Inhibitors, Anti-DLL4/NOTCH Antibodies [63, 64]. (OMP-21M18, REGN421, and MEDI0639 for anti-angiogenesis), γ-secretase inhibitors [65] (PF-03084014 inhibitor is generally safe and well tolerated by oral administration in advanced cancer). Wnt ligand inhibitors such as OMP-54F28 [66] which is a recombinant protein formed by the fusion of the immunoglobulin Fc to the CRD of FZD8 for blocked WNT, are undergoing clinical trials [67] despite being a promising strategy, it still has limitations such as the systemic toxicity of the antibodies used to block any of the pathways involved in the maintenance of CSC.CRISPR/Cas9 has become a powerful tool for changing the genome of many organisms. The open-label phase I study (NCT02793856) using CRISPR for cancer therapy was programmed cell death protein-1 (PD-1) knockout engineered. PD-1, a member of the CD28 superfamily of T-cell regulators expressed in a wide range of immune cells, including peripherally activated T cells, B cells, monocytes, NK cells, and DCs that consist of an Ig-V like extracellular domain, a transmembrane domain, and a cytoplasmic domain that harbors two tyrosine-based signaling motifs, interacts with two ligands [68]. These ligands, PD-L1 (CD274 or B7H1) and PD-L2 (CD273), were found expressed in some tumor cells. PD-L1 is expressed in many cell types such as vascular endothelium, reticular fibroblasts, non-mesenchymal stem cells, islet cells, astrocytes, neuronal cells, and keratinocytes. Interactions between the extracellular domains of PD-L1 and PD-1 attenuate T cell-activating signals and lead to inhibiting proliferation, survival, and production of growth factors such as EGF, TGF-β, and GM-CSF, and cytokines such as INFγ, TNF-α, IL-6 and IL-17. Activation of the PD-1/PD-L1 signaling pathway causes immunosuppression of T cell function, which is considered the main factor responsible for response immune escape [69]. However, cancer stromal cells can contribute to tumor microenvironment upregulates PD-L1 expression, by express GM-CSF and VEGF and promotes immune suppression. This effect is called “adaptive immune resistance”, because the tumor protects itself by inducing PD-L1 in response to IFN-γ produced by activated T cells. T cells ex vivo are evaluated for treating metastatic non-small cell lung cancer that has progressed after all standard treatments. Patients enrolled in the gene-editing trial provided peripheral blood lymphocytes and PD-1 knockout of T-cells by CRISPR/Cas9 performed ex vivo. The edited lymphocytes were selected, expanded and subsequently infused back into the patients. Four other trials applying the same concept of PD-1 knockout for treatment have been registred for other cancer types, including prostate, bladder, esophageal and renal cell cancer [62]. Recent studies propose as a target for colorectal cancer EGFR (overexpressed in 60–80% of aggressive tumors) or CAE as chimeric antigen receptors allow T-cells to recognize tumor cells and quickly destroy them [70]. This strategy is novel with safe and efficient results; mainly in hematological tumors with a lower response in solid tumors. New treatment approaches are still required since these present disadvantages such as side effects after their administration. In addition, it is still necessary to evaluate for prolonged periods if the resident tumor cells that do not evade this treatment by EMT are not able to develop metastasis. A recent report demonstrated that PD-L1 induces ZEB1, which activates OCT4 and Nanog signaling and upregulation of EMT on CSC. These promote chemoresistance and metastasis by increased phosphorylation of AKT and ERK, resulting in activation of the PI3K/AKT and MAPK/ERK pathways and an increase of MDR1 expression. Recently, Nivolumab, an anti-PD-L1 drug was approved for metastatic CRC resistant to fluoropyrimidine, oxaliplatin and irinotecan [68, 71]. This is because the therapeutic targets used are not specific to this cell population (CSC) and the pericytes, as the cells required to ensure the establishment of the metastases have not yet been taken into account.

Circular RNAs (circRNAs) are abundant and important members of the non-coding RNA family which are generally expressed at low levels and exhibit cell-type-specific and tissue-specific patterns, with an average half-life of 19–24 h and whose function remain mostly unknown [72]. There has recently been considerable attention on circRNA as a molecule that regulates or controls miRNA expression; therfore, they play a significant role in many fields of cancer biology. In tumor biology, circRNA emerges as an effective biomarker for the detection of cancers mainly because it allows differenciation between a normal cell and a tumor cell as well as exhibiting dynamic global changes in its expression levels during tumor progression [73]. In addition, because circRNA have normally been detected in saliva and blood, they can help as biomarkers that are able to predict sensitivity, the risk of metastasis or the prognosis of treatment. An example as a predictor of 5FU resistance, Xiong et al. identified three upregulated circRNAs (0007031, hsa_circ_0000504 and hsa_circ_0007006) in CRC by microarray analysis [74, 75]. However, until now they have not been used for therapeutic purposes. Also, the importance that these could have in colon cancer is unknown.

miRNAs are small 22-nucleotide non-coding RNAs that are distributed and abundant in almost all human tissue. They modulate hundreds of genes simultaneously and, therefore, control multiple signaling pathways involved in several processes such as apoptosis, proliferation, differentiation and migration [75]. Gene silencing by microRNAs occurs through imperfect/perfect complementary base pairing between a miRNA guide strand and the 3′ UTR region of the mRNA mainly; however, it has been detected that miRNAs bind to the 5′ UTR coding sequence as well as within promoter regions. The binding of miRNAs to the UTR region leads to translational repression or miRNA degradation [76] while miRNA interaction with the promoter region has been reported to induce transcription.

The dominant pathway by which miRNAs are processed begins with a pri-miRNA gene that is transcribed and processed by microprocessor complex and Drosha in the nucleus to form a pre-miRNA (precursor miRNA). Then pre-miRNA is exported to the cytoplasm via the activity of Exportin5/RanGTP-dependent manner and processed to produce the mature miRNA duplex by Dicer, AGO2 and TRBP, which are necessary components in the formation of the RNA-induced silencing complex (RISC). The RISC is then guided by the biological active strand to messenger RNA (mRNA) targets, which lead to gene silencing via mRNA degradation or translational inhibition [76].

During cancer initiation and progression, the expression levels of multiple miRNAs are aberrantly up or downregulated, resulting in an imbalance of cell pathways that reflect particular disease states associated with the regulatory response to chemotherapy, differentiation, proliferation, and migration in different malignancies which are useful for therapeutic purposes, and as diagnostic and prognostic biomarkers in cancer. Therefore, they may be strong weapons in the fight against chemoresistance in colon CSC. Gene expression studies have identified the clinical importance of miRNAs in pericytes or CSC on CRC. This is summarized in Table 1.

**Table 1:** Therapeutic approaches using, microRNAs against colon CSCs and pericytes [89, 90]

miRNA	Expression level	Target	Findings	Technique	References
21*	↑	ITGβ4	A prognostic tool, proliferation, invasion and metastasis	qPCR	10.4161/epi.26842 [91]
23a	↑	E-cadherin	Induced EMT process associated CRC metastasis	qPCR	10.1093/carcin/bgt274 [92]
	↓	ZO-1	Increasing vascular permeability and migration		10.3892/etm.2017.4972 [93]
24*	↑	Paxillin	Inhibited the killing effect of NK cells to colorectal cancer cells	qRT-PCR and Western blot	10.1016/j.biopha.2018.02.024 [94]
34a	↓	Inh3	Increased lymph node infiltration and metastasis in colon cancer patients	miRNA target prediction software miRWalk	10.1053/j.gastro.2017.04.017 [95]
126	↑	BCL-2 and p53	Potential tumour suppressor	qRT-PCR	10.1016/j.yexcr.2015.10.004 [96]
137	↓	TCF4	Suppresses cell proliferation, migration and invasion in colon cancer cell lines	RT-qPCR	10.3892/ol.2018.8364 [97]
143*	↑	IGF-IR	Inhibited cell proliferation, migration, tumor growth, angiogenesis and increased chemosensitivity to oxaliplatin treatment	qRT-PCR and western blot	10.4161/cc.24477 [98]
150	↑	c-Myb	Inhibits cell proliferation, induces cell apoptosis and inhibits cell migration and invasion in human CRC cells	qRT-PCR, western blot and DNA constructs and luciferase target assay	10.1111/jcmm.12398 [99]
200	↓	*ZEB1, ETS1* and *FLT1*	Increased EMT	TaqMan MicroRNA assays	http://doi.org/10.1136/gutjnl-2011-301846 [100]
203	↑	SOCS3	Potential for metastasis; promoted the differentiation of monocytes to M2 macrophages	RT-qPCR and Gene Set Enrichment Analysis (GSEA) TargetScan and miRanda	10.18632/oncotarget.20009 [101] 10.1177/1947601911425832 [102]
221	↑	RECK, RelA and STAT3	Migration and invasion in vitro and metastasis in vivo	qRT-PCR and western blot qRT-PCR and western blot	10.1016/j.febslet.2013.11.014 [103] 10.1053/j.gastro.2014.06.006 [104]
1246	↑	CCNG2	Promoted the proliferation, colony formation, invasion and migration, and inhibited the apoptosis	RT-qPCR and Dual luciferase reporter assay	10.3892/mmr.2015.4557 [105]

↑ = ; ↓ = *clinical trials

The effectiveness of microRNAS as nucleotide-based molecules has been compromised by inherent characteristics that they possess, such as: (1) stimulation of the innate immune system after induction of interferon responses; (2) inefficient binding due to a mutation in the sequence of the target mRNA; (3) short duration of the silencing effect, which requires high and sustained concentrations of payload in the target tissue. It also has other features such as serum instability due to rapid degradation by endo- and exonucleases in the bloodstream; inefficient cell entry inherent in the negatively charged nature of miRNA molecules, poor pharmacokinetic profile associated with a half-life of about 5 min, and rapid renal clearance due to their low molecular mass (≈ 13 kDa) [76–82] which can be overcome with efficient delivery systems. The properties of vector systems that can modify miRNA expression are briefly presented in Table 2 [83, 84].

**Table 2:** Vector systems

Vectors	Advantages	Disadvantages
Using VIRUS		
Adenovirus	↑ Efficiency and vector titers Insert capacity (max 8 Kb)	No integration Short-term expression ↑Immunogenicity
Adeno-associated virus	↑ Efficiency and vector titers ↓ Toxicity, no pathogenic ↓ Risk of mutagenesis Remains predominantly episomal	Requires helper virus to replicate Insert capacity (3-5 Kb)
Retrovirus	↓ Immune response in host Insert capacity (8 Kb) Integrates into genome	↓ Vector titers Incorpotates into dividing cells only Restricted tropism ↑ Risk of insertional mutagenesis
Lentivirus	Uptake in dividing and not dividing cells ↑ Insert capacity (8 Kb) Integrates into genome Next generation is self-inactiving for safe	↓ Vector titers Restricted tropism Risk of insertional mutagenesis
Non VIRAL		
Liposomes	Protect degradation by nucleasas Dose-dependent toxicity cationic polymers (PEI and PAMAM) ↓ Immune response in host rapid clearance from the bloodstream	Toxic effects on the liver and the kidney in mice ↓ Circulation half-life (minute–hours)
Nanoparticles	Protect degradation by nucleasas ↑ Circulation half-life (synthetic polymers sustained release over a period of days to several weeks) Dose-dependent toxicity ↑ Penetrability and solubility enhanced drug stability and biocompatibility facile synthesis and easy structural modification targeted drug delivery (specify and inespecify)	Toxic effects depends on the size and biodistribution
DNA nanostructures	Protect degradation by nucleasas Small size ↑ Precision and flexibility Non-toxic DNA nanostructures with their powerful structural control ↑ Biodistribution, biocompatibility	Localization and mapping of nanorobots in the human body are difficult using conventional optical microscopy techniques Effect desired require coordination collective nanorobots

Cationic polymers that are frequently used for intracellular delivery are polyethyleneimine (PEI) and polyamide amine dendrimers (PAMAM)

Encapsulating or protecting the microRNA by a vector with a reporter gene or cell tracking-dye allows evaluation of the activity in an in vivo model. A recent work evaluated an oral delivery system intended for treatment of colon cancer by encapsulating hSET1 antisense and SN38 anticancer in nanoparticles with results effective against HT29 cells. Also, more recently it was proposed against CRC to encapsulate miR-204-5p with poly (d, l-lactide-*co*-glycolide)/poly (l-lactide)-block-poly (ethylene glycol)-folate polymer to promote apoptosis and inhibit cell proliferation in an in vitro xenograft model with Luc-HT-29 [85–87]. Although it is a very promising area in the treatment against cancer, it still requires further evaluation of the role of different vectors to find the most suitable and safe, efficient and without long-term toxicity for its application in humans.

## Conclusions

As mentioned before, the important role that pericytes and tumor stem cells play in treatment resistance of patients with CRC makes these cells ideal candidates to limit tumor progression. Tumor suppressive microRNAs are potent molecules that might cure cancer. Recently, it was reported as advanced strategies for delivery of these microRNAs to the cell DNA-Doxorubicin against to HT-29 cells. Nano-sized DNA structures are of low cost, high stability, and feasible to synthesize. They are biosafe due to their lack of exogenous immune activity. Folic acid-DNA tetra-Dox strategy facilitates the targeted delivery of Doxorrubicin, enhances the anticancer HT-29 colon cancer efficiency of chemotherapy agent on colon cancer cells and provides a promising inspiration and idea for drug design [86, 88]. This delivery system is a very innovative and safe methodology; however, so far they have not been realized as a miRNA delivery system. That is why we believe that this therapeutic strategy could change the landscape of CRC.

## Data Availability

Not applicable.

## Abbreviations

CRC: colorectal cancer
ECs: endothelial cells
TME: tumor microenviroment
CSC: cancer stem cells
CTLs: cytotoxic T lymphocytes
TH: T helper cells
T-reg: T-regulatory cells
NK: natural killer
DCs: dendritic cells
MDSC: myeloid derived suppressor cells
IFNs: interferons
TNF: tumor necrosis factor
CAFs: cancer-associated fibroblasts
CXCL12: motif chemokine 12
CXCR4: chemokine receptor type 4
TGF- α: transforming growth factor alpha
TGF- β: transforming growth factor beta
PDGF: platelet-derived growth factor
FGF: fibroblast growth factor
CAF: cancer-associated fibroblast
IL-6: interleukin 6
MCP-1: monocyte chemoattractant protein 1
TNF-*α*: tumor necrosis factor alfa
DNA: deoxyribonucleic acid
VEGF: vascular endothelial growth factor
VEGFR: vascular endothelial growth factor receptors
SDF-1: stromall cell-derived factor 1
EMT: epithelial–mesenchymaltransition
Hh: Hedgehog
RNA: ribonucleic acid
ATOH1: atonal BHLH transcription factor 1
LGR5^+^: leucine-rich repeat-containing G-protein coupled receptor 5
ALDH1: aldehyde dehydrogenase
SMA: smooth muscle actin
EGF: epidermal growth factor
EGFR: epidermal growth factor receptor
CD: cluster of differentiation
HER: human epidermal growth factor receptor
KLF4: Kruppel-like factor 4
Bmi-1: polycomb complex protein
PTEN: phosphatase and tensin homolog gene
ECM: extracellular matrix
MSC: mesenchymal stem cell
PDGFR- β: platelet-derived growth factor receptor-beta
EPT: epithelial to pericyte transition
KRAS: Ki-ras2 Kirsten rat sarcoma viral oncogene homolog
NF-κB: nuclear factor kappa-light-chain-enhancer of activated B cells
ADC: antibody drug conjugate
PD-1: programmed cell death protein-1
PD-L1: programmed death-ligand 1
PD-L2: programmed death-ligand 2
ZEB1: Zinc Finger E-Box Binding Homeobox 1
MDR1: multidrug resistance protein 1
EBV: Eppstein–Barr virus
CAR: chimeric antigen receptor
TRAC: T cell receptor α chain
CMD: carboxymethyl dextran
PEI: polyethyleneimine
PAMAM: polyamide amine dendrimers
